# Electrochemical Properties of Homogeneous and Heterogeneous Anion Exchange Membranes Coated with Cation Exchange Polyelectrolyte

**DOI:** 10.3390/membranes9010013

**Published:** 2019-01-11

**Authors:** Xenia Nebavskaya, Veronika Sarapulova, Dmitrii Butylskii, Christian Larchet, Natalia Pismenskaya

**Affiliations:** 1 Kuban State University, 149 Stavropolskaya st., 350040 Krasnodar, Russia; vsarapulova@gmail.com (V.S.); dmitrybutylsky@mail.ru (D.B.); n_pismen@mail.ru (N.P.); 2 Institut de Chimie et des Matériaux Paris-Est, UMR7182 CNRS—Université Paris-Est, 2 rue Henri Dunant, 94320 Thiais, France; larchet@u-pec.fr

**Keywords:** ion exchange membrane, polymer, membrane modification, current–voltage curve, limiting current

## Abstract

Coating ion exchange membranes with polyelectrolyte has been proven to be a cheap way to reduce concentration polarization and increase limiting current (for polyelectrolytes carrying fixed groups of the same sign of charge with respect to the membrane bulk), to create high monovalent selectivity, and to add the function of H^+^/OH^−^ ions generation (for polyelectrolytes bearing fixed groups of the opposite sign of charge with respect to the membrane bulk). In the latter case, the balance between the counterion transport and the H^+^/OH^−^ ions generation is affected by parameters of the substrate and the modifying layer. In this study we investigated the electrochemical characteristics of homogeneous Neosepta AMX-Sb and heterogeneous MA-41P membranes coated with one, two, or three layers of oppositely charged polyelectrolyte (the maximum thickness of each layer was 5 µm). It was found that the limiting current decreased earlier and the generation of H^+^/OH^−^ ions was stronger in the case of the heterogeneous membrane. The shift in the pH of the solution depended more on the generation of H^+^/OH^−^ ions at the modifying layer/solution interface than on the generation at the membrane/modifying layer interface, and in all cases water splitting started in the same range of potential drops over the membrane.

## 1. Introduction

The modification of ion exchange membrane (IEM) surfaces with a polyelectrolyte layer was first proposed by Sata et al. [1], who aimed to improve monovalent selectivity through the introduction of a selective layer carrying fixed groups oppositely charged with respect to the fixed groups of membrane bulk. In practice, the samples with functional groups of modifying layers being oppositely charged to the groups in membrane bulk became bipolar membranes with two functions: desalination and H^+^/OH^−^ ions generation [2]. The great role in the electrochemical properties of such IEM is played by the bipolar junction between the membrane and the modifying layer, at which the H^+^/OH^−^ ions generation occurs.

Further studies broadened the range of application of layered electrochemical materials. Membrane-coated electrodes attracted interest for various electrochemical applications [3,4,5]. In the case of IEM, for redox flow batteries, layered polyelectrolyte composite membranes showed good conductivity and low permeability to vanadium ions [6], and a thorough review of methods to achieve high monovalent selectivity of IEM in the separation processes (including several techniques for coating surfaces with charged and uncharged layers) was recently published [7]. Surface modification of ion exchange membranes with polyelectrolytes was used for pH correction [8], for suppression of fouling [9,10] or scaling [11], and for facilitation of the electro-osmotic slip of water near the surface [12]. It was shown that excellent monovalent selectivity can be achieved for IEM using layer-by-layer coating with polyelectrolytes carrying functional groups of alternating charges [13,14], or even for multilayers applied at uncharged porous supports [15]. For these systems, very high separation factors and adequate electric resistance were reported. However, several possible additional properties of layered IEM, such as the ability to generate H^+^ and OH^−^ ions, should be taken into account for possible applications.

The balance between the transport of salt ions and the generation of H^+^/OH^−^ ions depends on the thickness of the applied layer. For asymmetric bipolar membranes produced by the pouring and spreading of relatively thick modifying layers, this dependence was studied by Zabolotsky et al. [8], who found that the water splitting increases (and salt ion transport decreases) with the layer thickness. For thinner layers, the details of such dependency are not yet known, hence one of the tasks of our study was to determine the effect of modifying-layer thickness on the electrochemical properties (limiting current density of salt counterion and ability for H^+^/OH^−^ ions generation) of resulting membranes in the cases of applied layers with low thickness.

Another question not yet answered was the role of the electrical heterogeneity of membrane support. Heterogeneous membranes are cheaper than homogeneous ones, hence they can be used for the design of cheaper novel materials with improved monovalent selectivity and reasonable electrochemical properties. What makes the difference between the homogeneous and the heterogeneous membranes in this case is that the appearance of nonconductive zones at the surface of heterogeneous membranes make the current lines concentrated on conductive zones [16]. As a result, the local current density is higher on conductive zones, which causes stronger concentration polarization and water splitting. Modified homogeneous membranes [13] and modified heterogeneous membranes [8] were studied separately, but the differing methods of modification and testing protocols do not allow direct comparison of results. To study the effect of this difference, we compared the properties of two series of membranes based on supporting membranes differing in homogeneity. The homogeneous supporting membrane was produced by the paste method (Neosepta AMX-Sb) and the heterogeneous supporting membrane was produced by hot rolling (MA-41P).

## 2. Materials and Methods

### 2.1. Membranes and Materials

All samples in this study were prepared from the stock of commercial MA-41P (Shchekinoazot, Russia) and Neosepta AMX-Sb (Astom, Tokyo, Japan) membranes purchased from their respective companies. Both membranes contained an ion exchange matrix of the same composition, poly(styrene-divinylbenzene) copolymer, which carried quaternary ammonium bases (Table 1). They differed in their degrees of heterogeneity.

Data from manufacturers and from previous studies described the procedures of the commercial production of MA-41P and AMX-Sb as follows: During the production of the MA-41P, a mixture of powdered ion exchanger and powdered polyethylene was hot rolled between two layers of Nylon 6 reinforcing cloth. As determined from analysis of the surface fraction of the ion exchanger (based on scanning electron microscopy visualizations of Russian heterogeneous membranes in top view), only about 28% of the resulting membranes can conduct electrical current, even in a swollen state [17]. During the production of AMX-Sb, a mixture of finely powdered polyvinyl chloride and functionalized monomer was applied to reinforced polyvinyl chloride cloth, then heated for polymerization [18], so that the dimensions of heterogeneities were far smaller than in the case of the MA-41P [19] and, before coating, almost 100% of the commercial AMX-Sb membrane was electrically conductive [20].

Commercial membranes used in this study were chosen based on their broad use for electrodialysis. The AMX-Sb homogeneous anion exchange membrane (AEM) operating in electrodialyzers [23] is recommended by its manufacturer for a number of applications including separation of monovalent ions. In Reference [24], this type of AEM is cited as widely used in ED for food industry applications. The MA-41P belongs to the MA-41 series of membranes, which is currently the standard Russian heterogeneous AEM routinely used both for studies and in industry [25,26,27]. The MA-41P membrane is known for its mechanical stability and durability. Coupled with its low cost, these properties make the MA-41P membrane promising for the production of modified membranes [28].

**Figure 1:**
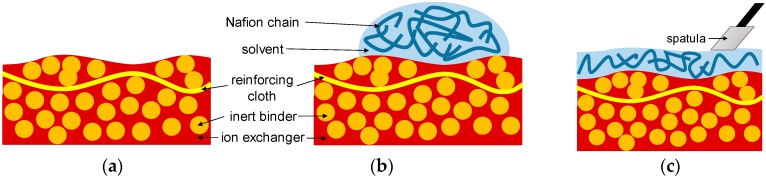

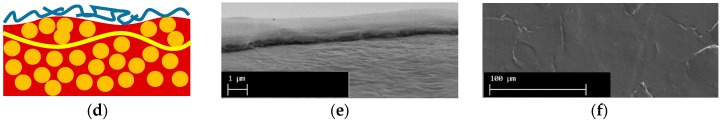
Sketch of the process of modification (**a**–**d**) and fragments of SEM images of resulting membranes. (**a**) The commercial membrane to be subjected to modification (the sketch illustrates the case of AMX-Sb); (**b**) the modifying dispersion is applied; (**c**) it is manually spread with a spatula; (**d**) the solvent evaporates, leaving the relatively thin modifying layer. (**e**) A fragment of an SEM image portraying a cross section of MA-41P membrane modified with one layer of polyelectrolyte; (**f**) A fragment of an SEM image showing a top view of the same membrane.

Prior to modification, the membranes were equilibrated with isopropyl alcohol. The dispersion of Nafion was used for coating. Nafion used in this study was perfluorinated ionomer, which contains sulfonic groups. To produce the modifier, a 5% (wt.) commercial dispersion of ionomer in water and alcohols bought from Sigma-Aldrich was diluted fivefold with isopropyl alcohol. The resulting dispersion was spread to produce the modifying layer as thin as possible (differing here from the approach used to produce the asymmetric bipolar membranes with relatively thick modifying layers [8]) onto the 5 cm × 5 cm samples fixed to glass; the membranes were then left to dry in air for 24 h. For spreading, the selected volume (0.25 mL in this case) was applied at the center of the samples and fixed to glass (which rests at the level surface), then the dispersion was manually spread onto the membrane with a spatula (Figure 1) and left to dry.

Previous preparation of the modified membranes following this protocol showed that the thickness of the single layer applied to homogeneous membranes does not exceed 5 µm [29]. After that, the membranes were soaked in concentrated (6.15 M, or 36%) NaCl solution, and every 2 h the solution was diluted two-fold. After four such dilutions, the membranes were transferred into 0.02 M NaCl solution and left for 16 h.

It should be noted that, according to the row of catalytic activity of functional groups [30], neither sulfonic groups of Nafion nor quaternary ammonium bases of AMX-Sb/MA-41P boost the H^+^/OH^−^ ions generation reaction.

The thicknesses were measured for swollen commercial membranes and swollen modified membranes with a micrometer. However, it was found that the modification did not lead to a detectable change in thickness, and that the thickness of commercial AMX-Sb and all composite membranes based on it were in range of 134 ± 3 µm, while thickness of commercial MA-41P and all composite membranes based on it were in the range of 545 ± 3 µm (Table 1). Visualizations of cross sections of MA-41P membranes modified with a single layer of polyelectrolyte (Figure 1e) showed that the thickness of dry layers applied to heterogeneous membranes was about 1 µm.

### 2.2. Current–Voltage (I–V) Curves

**Figure 2:**
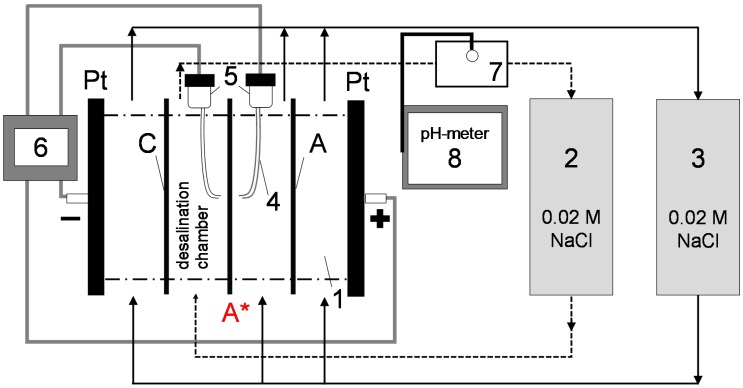
Principal scheme of the flow-through four-chamber electrodialysis cell (**1**) with an anion exchange membrane (AEM) under study (A*); auxiliary cation exchange (**C**) and anion exchange (**A**) membranes; tanks with 0.02 M NaCl solutions (**2,3**); Luggin capillaries (**4**) with Ag/AgCl electrodes (**5**); an Autolab PGSTAT N100 power source/voltmeter (**6**); and a pH-sensitive electrode (**7**) connected to a Mettler Toledo SevenCompact S220 pH meter (**8**). Adapted from [32].

Current–voltage (I–V) curves of studied samples were obtained using a flow-through 4-chamber cell (Figure 2). In all cases tested, the AEM formed a desalination chamber with a Neosepta CMX cation exchange membrane. On the other side, the concentration chamber was separated from the electrode chamber by the MA-41P membrane. The operating area was 2 cm × 2 cm, the intermembrane distance was 0.65 cm, and the operating solution was 0.02 M NaCl pumped at a flow rate of 0.46 cm/s. The polarizing electrodes were made of polished platinum 2 cm × 2 cm and the measurement electrodes were closed Ag/AgCl electrodes connected to Luggin capillaries, the tips of which were located at both sides of the studied membrane against its geometrical center. The distance from the surface was around 0.5 mm. The setup was laboratory-assembled and based on the setup described in more detail in [31,32], with several changes in equipment.

I–V curves were obtained in galvanodynamic mode. The current density was swept from 0 to 6.0 mA/cm^2^, increasing stepwise at 2.5 µA/cm^2^ per second. Autolab PGSTAT N100 was used both to create the current sweep and to register the potential drop over the membrane. The theoretical limiting current density was calculated by the Lévêque equation [33]:

jlimtheor=z1C1FDh(T1−t1)[1.47(h2V0LD)13−0.2],

where *z*_1_ and *C*_1_ are the charge and molar concentration of counterion in solution bulk, respectively; *F* is the Faraday constant; *D* is the diffusion coefficient of salt in solution; *h* is the intermembrane distance; *T*_1_ and *t*_1_ are the counterion transport number in the membrane and solution, respectively; *V*_0_ is the linear solution pumping rate; and *L* is the length of the desalination path. The *D* value was taken equal to that at infinite dilution and 25 °C, which is 1.61 × 10^−9^ m^2^/s. For the experimental conditions, *T*_1_ was assumed to be equal to 1 (since the membranes are highly selective for counterions in such dilute solutions, and at currents close to the limiting current density the input of water splitting is found to be negligible), *t*_1_ to 0.603, *h* to 6.3 mm, and *L* to 2.0 cm. The calculated limiting current density was 3.12 mA/cm^2^.

**Figure 3:**
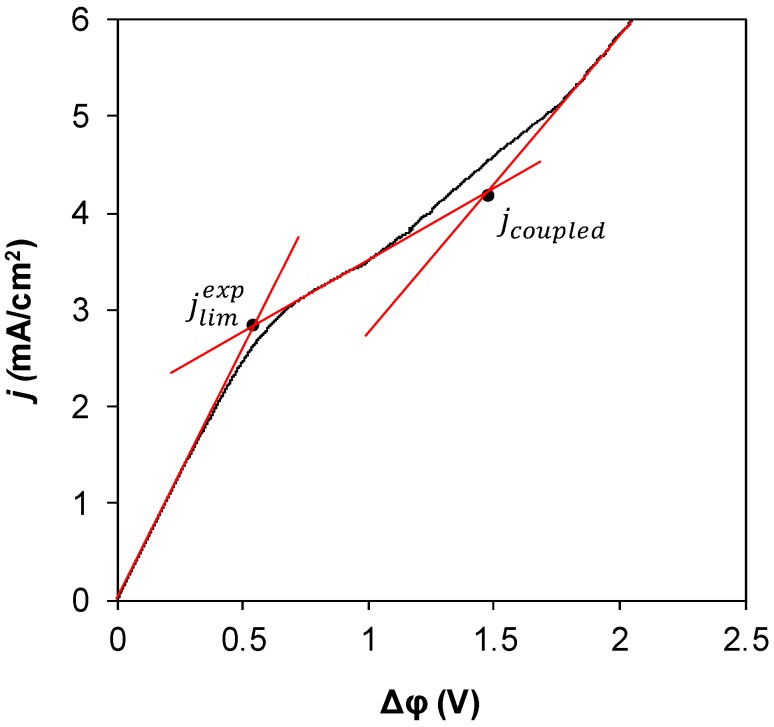
Determination of the experimental limiting current and the current at which the coupled effects of concentration polarization arise. The resistance of the membrane can also be determined from the slope of the tangent drawn to the initial part of the curve.

To determine such parameters as experimental limiting current density and the current at which the coupled effects of concentration polarization arise, the following procedure was employed:

The I–V curves were built in Δφ vs. *j* coordinates, where Δφ was the experimental potential difference registered between Luggin capillaries and *j* was the experimental current density (Figure 3). Then, the tangents were drawn to the initial section of the I–V curve (the so-called ohmic region), to the initial section of the plateau region and to the end of the overlimiting currents region. The slope of the first tangent depends on the resistance of the membrane. It shows if the resistance changed due to modification. The intersection of tangents drawn to the ohmic region and to the plateau region gives the experimental limiting current. The intersection of tangents drawn to the plateau region and to the overlimiting currents region gives the point where the transition to the overlimiting current range occurs.

In all cases, the difference in pH between the outlet and the inlet of the desalination chamber was registered together with the I–V curves with a Mettler Toledo SevenCompact S220 pH meter (Shanghai, China). Before the theoretical limiting current was reached, pH value was recorded each 5 min (starting at the beginning of the experiment at 0 min). When the limiting current was reached and the water dissociation reaction was expected to intensify, the lap between the measurements was reduced to 2 min. Since the cation exchange membrane in all tests was the same (strongly acidic Neosepta CMX), the pH-metry data were used to evaluate the relative rate of generation of H^+^ and OH^−^ ions on the AEM.

## 3. Results and Discussion

**Figure 4:**
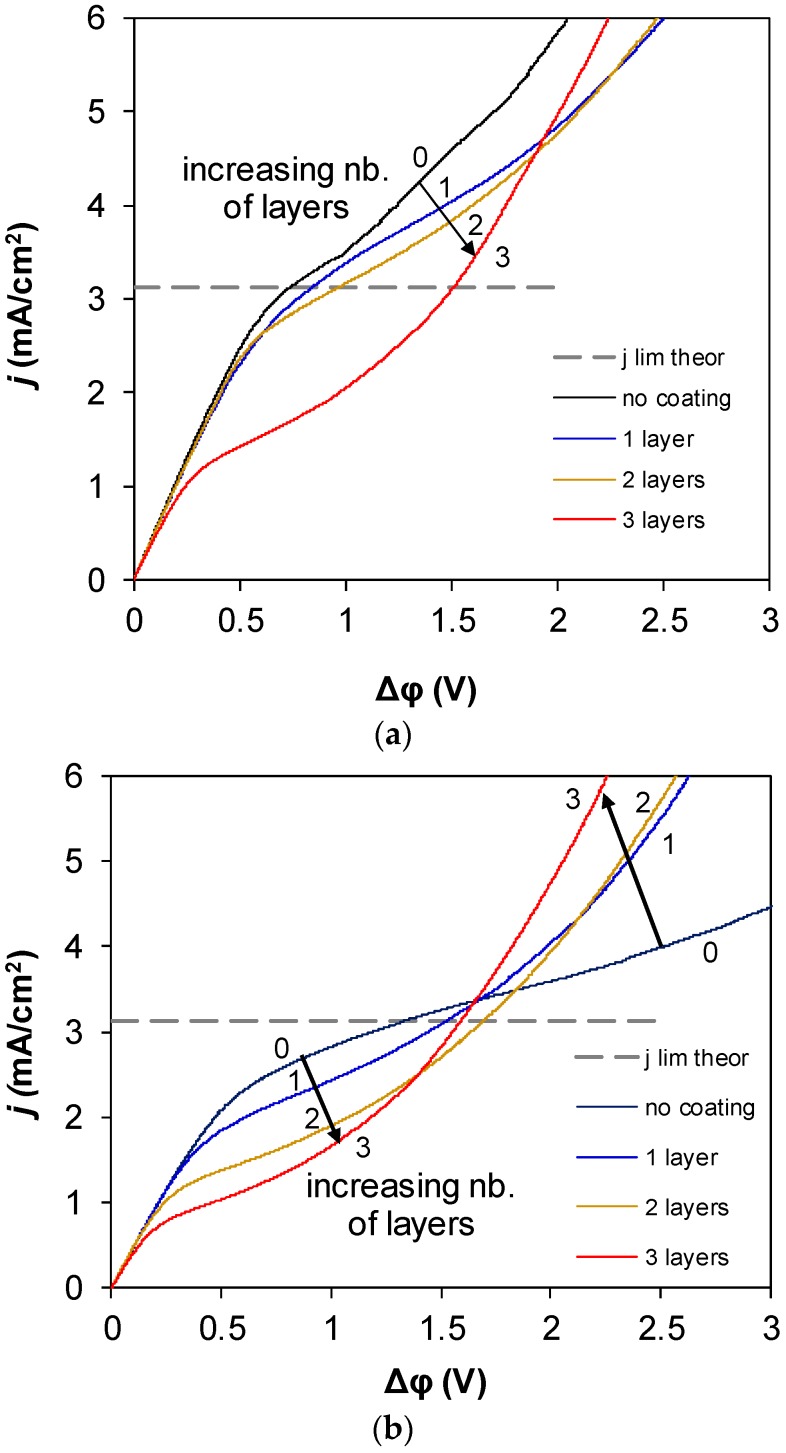
I–V curves of the homogeneous AMX-Sb membrane and its modifications (**a**) and the heterogeneous MA-41P membrane and its modifications (**b**). Arrows show the increased number of changes occurring with the additional layers of Nafion applied. The dashed line shows the theoretical limiting current calculated by the Lévêque equation. Numerals denote the number of applied layers ranging from zero (no coating, i.e., the commercial membrane) to three.

Figure 4 gives the I–V curves of studied membranes. It should be noted that even the experimental limiting current of the commercial MA-41P heterogeneous membrane was lower than the theoretical limiting current calculated by the Lévêque equation. This can be explained by a higher local current density at the conductive zones due to the surface being partially occupied by nonconductive material, leading to higher concentration polarization and a faster decrease of salt concentration near the membrane surface.

Comparison of the I–V curves that were measured for homogeneous and heterogeneous membranes showed that in general the changes that occurred with electrochemical properties (due to their modification with polymer carrying the functional groups oppositely charged to groups in membrane bulk) were similar. However, there were also peculiarities.

Firstly, it can be seen that in all cases the experimental limiting current density decreased with the increasing number of layers. Such changes are typical for monopolar ion exchange membranes transforming into bipolar membranes due to the increasing thickness of the oppositely charged layer [8] when the bipolar junction blocks the transport of salt ions.

**Figure 5:**
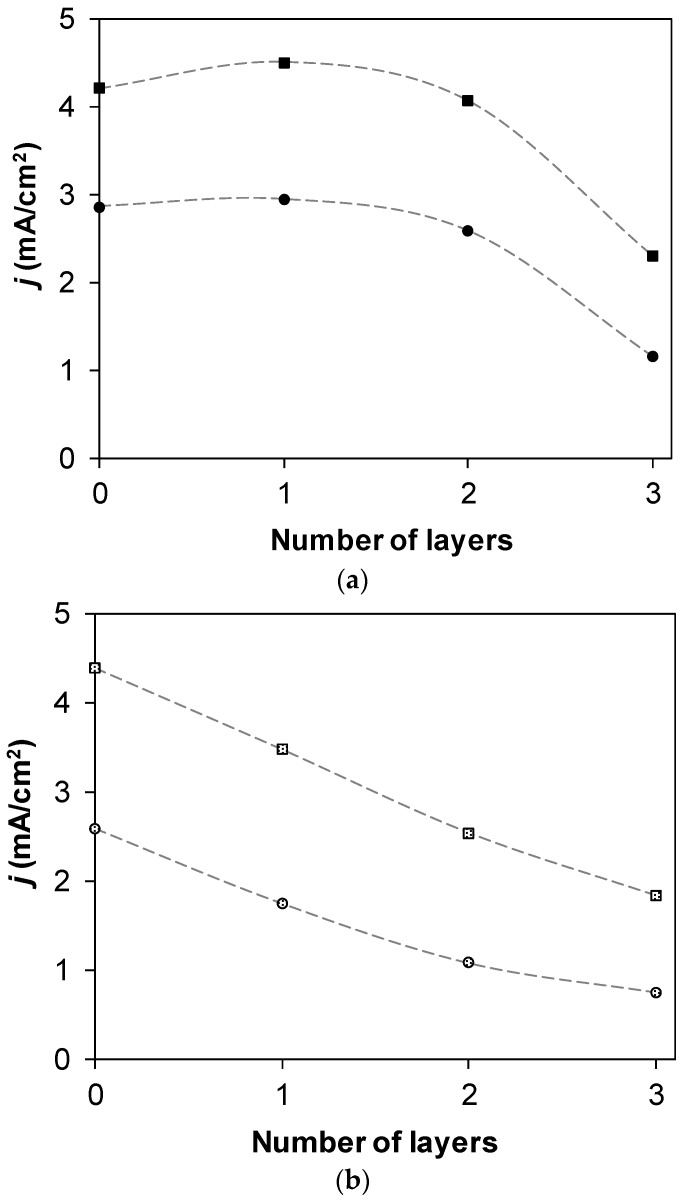
Dependence of experimental limiting current densities ($i_{lim}^{exp}$, circles) and current densities of transition to the overlimiting current range (*i*_coupled_, squares) on the number of applied layers of polyelectrolyte for AMX-Sb (**a**) and MA-41P (**b**).

Secondly, in all cases, the differential resistance of membranes, which could be determined from the slope of the I–V curve in the underlimiting currents region, did not strongly change with the application of layers. However, the character of changes that occurred with modifications of the AMX-Sb membrane differed from the character of changes that occurred with modifications of the MA-41P membrane. Properties of the MA-41P changed gradually (the experimental limiting currents and currents at which the overlimiting current range starts to decrease step by step with each newly applied layer), while properties of the commercial AMX-Sb and samples modified with one or two layers of polymer only slightly differed, and the significant decline occurred only after three layers were applied (Figure 5).

Data of pH-metry showed how water splitting was balanced in the desalination chamber between a cation and an anion exchange membrane. The H^+^ ions produced at the AEM and the OH^−^ ions produced at the cation exchange membrane entered the desalination chamber, and acidification of desalted solution provided evidence that the reaction occurred more strongly at anion exchange membranes, while alkalification of desalted solution showed that the reaction occurred more strongly at cation exchange membranes [34]. Since the cation exchange membrane (Neosepta CMX) was the same in all measurements, the difference in pH between the exit and the entrance of the desalination chamber could be used to compare the intensity of water splitting at different anion exchange membranes.

**Figure 6:**
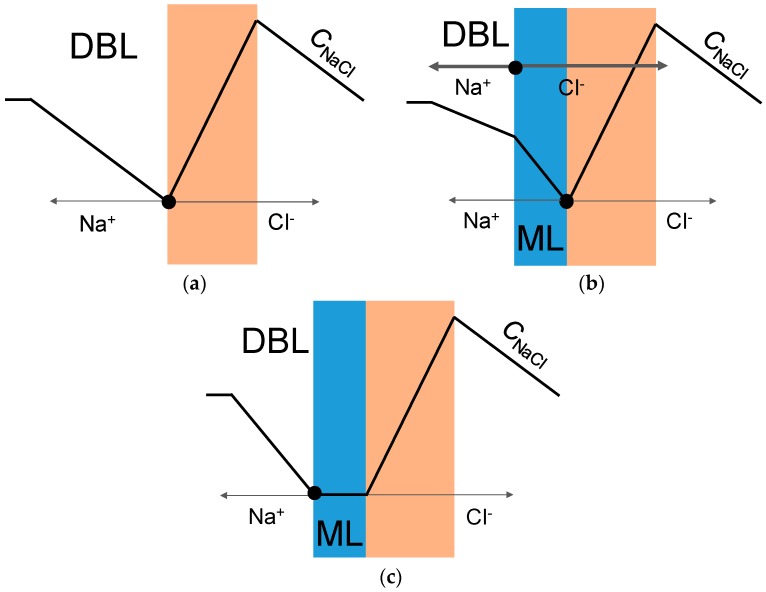
Limiting states reached at the commercial membrane (**a**), at the modifying layer/substrate membrane boundary of the modified membrane (**b**) and at the modifying layer/desalted solution boundary of the modified membrane (**c**). Pink zone denotes the substrate membrane, DBL denotes the (depleted) diffusion boundary layer and ML denotes the modifying layer. Black lines show the concentration profile of salt and grey lines show the fluxes of salt ions.

In the case of commercial monopolar membranes, it is known [34] that the generation of H^+^/OH^−^ ions occurs only at the membrane/desalted solution boundary. In the case of monopolar membranes modified with polyelectrolyte carrying the fixed groups with the charge opposite to membrane bulk, the reaction can occur both at the substrate membrane/modifying layer (bipolar junction) or at the modifying layer/desalinated solution boundaries (Figure 6). The electrolyte concentration decreases faster at the bipolar junction since the delivery of salt ions to this interface is hindered from both directions by electrostatic repulsion forces, meaning that the limiting state is reached faster at this boundary. This first limiting state corresponds to the experimental limiting current. With further increase of potential drop, two situations are possible: either growing potential drop will cause increased transport of salt ions by the electromigration mechanism (despite counterions for the substrate membrane being co-ions for the modifying layer), or growing potential drop will cause intensive H^+^/OH^−^ ions generation at the bipolar junction.

**Figure 7:**
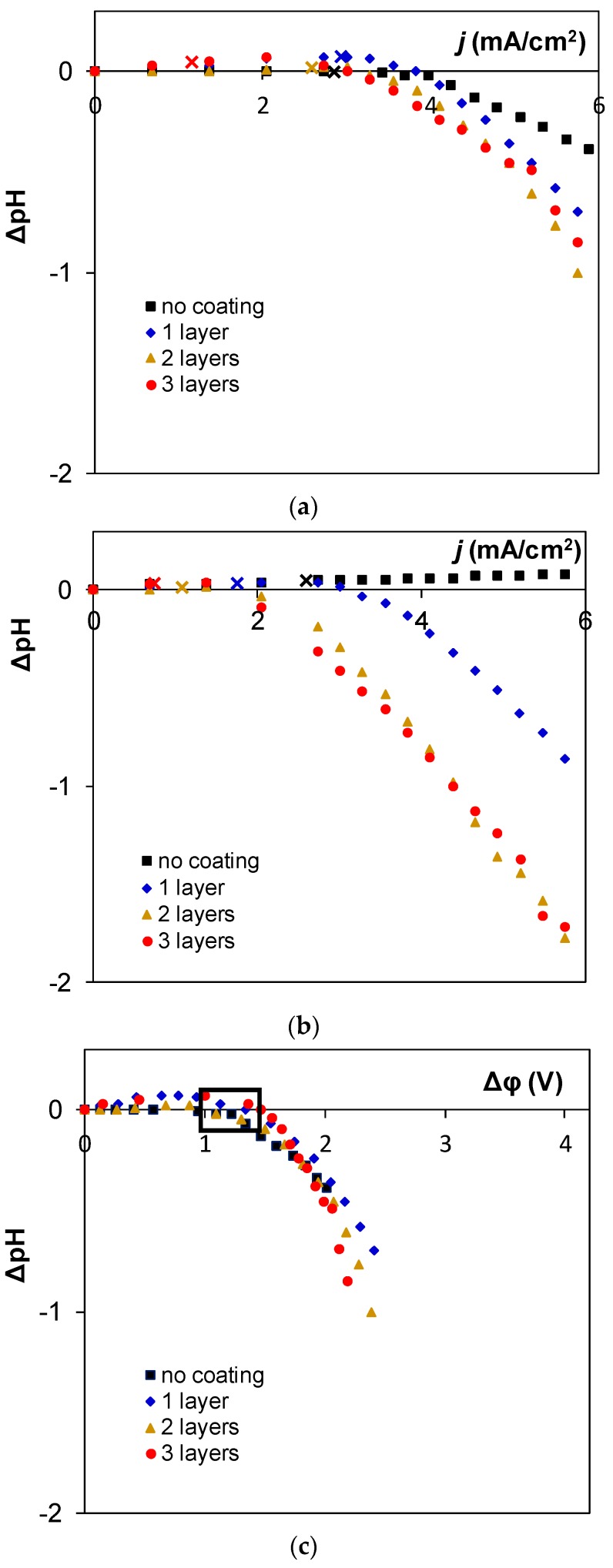

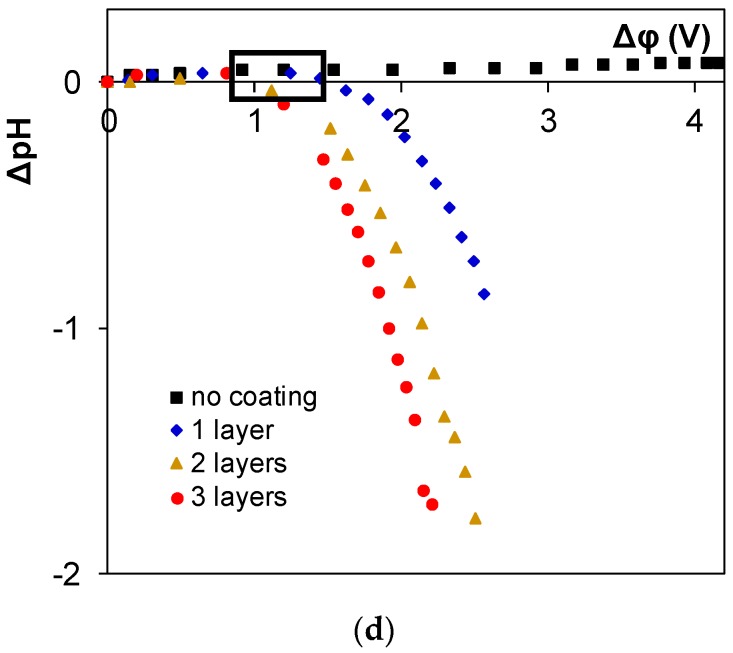
Dependence of the difference in pH between the exit and entrance of the desalination chamber formed by CMX cation exchange membranes and AEMs based on AMX-Sb (**a**,**c**) and MA-41P (**b**,**d**) on current density (**a**,**b**) or potential drop over the membrane (**c**,**d**). Crosses show the approximations of pH made for experimental limiting current densities based on pH values registered for the two closest current densities. Squares denote the commercial membranes, diamonds represent the membranes with one layer of coating, triangles represent the membranes with two layers of coating, and circles represent the membranes with three layers of coating.

The experimental pH-metry data are given in Figure 7. Dependences of pH difference are given in Figure 7a,b together with experimental limiting current values, and it can be seen that intensive water splitting started much later than the limiting currents were reached. In these cases, therefore, the bipolar junction did not determine the beginning of H^+^/OH^−^ ions generation. In all cases water splitting started in the same relatively small window of potential drops over the membrane (denoted by empty black boxes in Figure 7c,d), and the corresponding current value decreased only due to increasing resistance from the system. This dependence of critical potential drop on the number of layers shows that the desalted solution/modifying layer boundary plays a more important role in development of H^+^/OH^−^ ions generation.

The registered pH values (Figure 7) show the common trend between the homogeneous and heterogeneous membranes: the H^+^/OH^−^ ions generation increased with the number of applied layers, while water splitting was much stronger in the case of modified membranes based on the heterogeneous MA-41P membrane than modified membranes based on the homogeneous AMX-Sb membrane.

**Figure 8:**
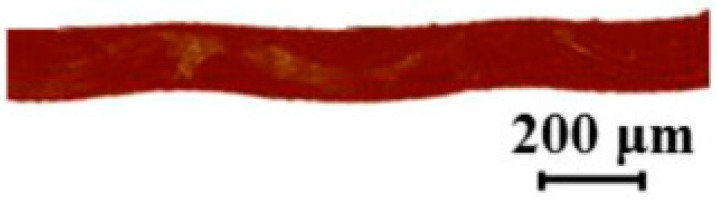
Cross section of swollen AMX-Sb membrane. Adapted from [20].

A hypothesis can be suggested regarding the differences in behavior of AMX-Sb and MA-41P. It was previously concluded from the peculiarities of electrochemical properties of the AMX-Sb membrane modified with Nafion that the singular application of modifier leads to formation not of a continuous and even layer, but of island-like structures [29]. This is further supported by the geometrical heterogeneity of the homogeneous membrane produced by Astom, which does not have a flat surface but instead possesses repeating hills and valleys [20,35] (Figure 8), where the modifier might be accumulated in the valleys. Since the application of a second layer does not lead to such big changes in the I–V curve, it might be suggested that, like coating with the first layer, the second-layer coating does not lead to complete continuous coverage, while coating with a third layer might lead to full coverage.

Smaller limiting currents and stronger water splitting found for modified membranes based on MA-41P might be explained by the heterogeneity of MA-41P—according to [17], only about 28% of the surface of MA-41 series membranes is represented by ion exchanger. This causes higher current density at conductive areas and, as a result, higher concentration polarization and more pronounced coupled effects—which in this case, judging by Figure 7, were represented by water splitting. Additional factors that may play a role in the difference are the possible preferable sorption of polyelectrolyte on the grains of ion exchanger (due to electrostatic interaction between the oppositely charged fixed groups) and lessened sorption on (uncharged) polyethylene. This would cause the thickening of the layer on the conductive zones in comparison with a homogeneous surface, meaning that such materials might benefit from a decrease in the amount of added modifier.

## 4. Conclusions

It was shown that spreading one or two thin layers of polymer, with functional groups oppositely charged with respect to the ones in membrane bulk, onto the surface of commercial homogeneous AMX-Sb membrane resulted in the creation of new membranes with resistance and limiting current comparable with the original one. As such, this method is suitable for the creation of materials that combine good transport properties of supporting membrane with monovalent selectivity, due to the oppositely charged layer that was applied. The application of a larger number of layers or the use of heterogeneous membrane led to a decrease of the limiting current and hastening of the onset of overlimiting mode, accompanied by growing H^+^/OH^−^ ions generation. In all cases, the H^+^/OH^−^ ions generation started in the same range of potential drops and this reaction was more active at the external (modifying layer/desalted solution) boundary than at the internal (modifying layer/substrate membrane) boundary. It was found that H^+^/OH^−^ ions generation was stronger, and that the limiting current decreased more readily, in the case of a heterogeneous membrane. Such peculiarities would require special attention if the heterogeneous membranes were to be used for the production of novel modified membranes.

## Figures and Tables

**Table 1:** Properties of AMX-Sb and MA-41P commercial substrate membranes.

Property	AMX-Sb	MA-41P
Ion exchanger	poly(styrene-divinylbenzene) copolymer which carries quaternary ammonium bases	
Diameter of ion exchange grains	continuous phase	5–40 µm [17]
Reinforcing material	PVC grains up to 100 nm in diameter [21] *; PVC cloth	continuous phase of PE; Nylon 6 cloth [22]
Ion exchange capacity, mM/g (swollen sample)	1.28 ± 0.05	1.25 ± 0.05
Thickness, µm (swollen sample)	134 ± 3	545 ± 3
Resistance in 0.02 M NaCl solution (calculated from current–voltage curves), Ohm	46	52

* From visualization of the surface of cation exchange membranes prepared by the same method.
